# Human Borna disease virus 1 encephalitis shows marked pro-inflammatory biomarker and tissue immunoactivation during the course of disease

**DOI:** 10.1080/22221751.2022.2098831

**Published:** 2022-07-27

**Authors:** Jessica Rauch, Johanna Friederike Steffen, Birgit Muntau, Jana Gisbrecht, Kirsten Pörtner, Christiane Herden, Hans Helmut Niller, Markus Bauswein, Dennis Rubbenstroth, Ute Mehlhoop, Petra Allartz, Dennis Tappe

**Affiliations:** a Bernhard Nocht Institute for Tropical Medicine, Hamburg, Germany; b Department of Infectious Disease Epidemiology, Robert Koch Institute, Berlin, Germany; c Institute for Veterinary Pathology, Justus-Liebig-University Gießen, Gießen, Germany; d Institute of Clinical Microbiology and Hygiene, Regensburg University Hospital, Regensburg, Germany; e Friedrich-Loeffler-Institut, Federal Research Institute for Animal Health, Greifswald-Insel Riems, Germany

**Keywords:** BoDV-1, bornavirus, microglia, astrocyte, cytokine, chemokine

## Abstract

Human Borna disease virus 1 (BoDV-1) encephalitis is a severe emerging disease with a very high case-fatality rate. While the clinical disease, case definitions, diagnostic algorithms and neuropathology have been described, very little is known about the immunological processes of human BoDV-1 encephalitis. Here, we analyzed serum and cerebrospinal fluid (CSF) samples from 10 patients with fatal BoDV-1 encephalitis for changes of different cytokines, chemokines, growth factors and other biomarkers over time. From one of these individuals, also autoptic formalin-fixed brain tissue was analyzed for the expression of inflammatory biomarkers by mRNA levels and immunostaining; in a further patient, only formalin-fixed brain tissue was available and examined in addition. A marked and increasing immune activation from the initial phase to the last phase of acute BoDV-1 encephalitis is shown in serum and CSF, characterized by cytokine concentration changes (IFNγ, IL-5, IL-6, IL-9, IL-10, IL-12p40, IL-13, IL-18, TGF-β1) with a predominantly pro-inflammatory pattern over time. IFNγ production was demonstrated in endothelial cells, astrocytes and microglia, IL-6 in activated microglia, and TGF-β1 in endothelial cells, activated astrocytes and microglia. This was paralleled by an increase of chemokines (CCL-2, CCL-5, CXCL-10, IL-8) to attract immune cells to the site of infection, contributing to inflammation and tissue damage. Pathologically low growth factor levels (BDNF, β-NGF, PDGF) were seen. Changed levels of arginase and sTREM further fostered the pro-inflammatory state. This dysbalanced, pro-inflammatory state likely contributes importantly to the fatal outcome of human BoDV-1 encephalitis, and might be a key target for possible treatment attempts.

## Introduction

Human bornavirus encephalitis is a severe disease caused by two related zoonotic members of the *Bornaviridae* family, the rarely detected variegated squirrel bornavirus 1 (VSBV-1; species *Mammalian 2 orthobornavirus*) and the emerging Borna disease virus 1 (BoDV-1; species *Mammalian 1 orthobornavirus*). While human VSBV-1 infection is so far limited to five confirmed cases in private holdings and zoological gardens, and acquired through contact to exotic squirrels and their secretions [1–6], human BoDV-1 encephalitis cases are increasingly detected and have been described in regions endemic for animal Borna disease (BD) in Germany [6–14]. Besides these sporadic cases, a single solid organ transplantation-related cluster of BoDV-1 encephalitis has also been reported, originating from a donor living in a BoDV-1 endemic region [7]. By now, there are around 40 known human cases in Germany, either published or notified to the Robert Koch Institute [13]. Most of them were diagnosed retrospectively from archived brain tissue material. The geographic presence of BoDV-1 extends to endemic parts of Switzerland, Austria, and Liechtenstein [15,16], and the virus is harboured at least by the bicoloured white-toothed shrew (*Crocidura leucodon*) as a natural reservoir [15,17].

Human BoDV-1 encephalitis has a very high case-fatality rate [6,18] and its clinical course seems to be more rapid than the course of VSBV-1 encephalitis [1,2,6,8–10]. Clinically, after a short unspecific flu-like prodromal period often with headache and fever, patients show neurological signs including confusion, psychomotor slowing, ataxia or epileptic seizures. The disease rapidly progresses with deterioration in consciousness leading to deep coma within days and death after several weeks [8–10,12]. BoDV-1 is non-cytopathogenic, strongly cell-bound and neurotropic in accidental hosts such as humans. Besides neurons, also astrocytes and oligodendrocytes can be infected. For both known zoonotic bornaviruses, a non-purulent panencephalitis can be demonstrated histopathologically, characterized by malacia, perivascular B and T cell cuffing, as well as macrophage accumulation, CD4+ and CD8+ T cell parenchymal infiltration, strong microglial activation, and partly bizarre reactive astrocyte changes [9,11,19]. For human VSBV-1 encephalitis immunopathological mechanisms have already been shown [19]. While the clinical disease [6–14], cranial magnetic resonance imaging findings [7–10,13,20], case definitions and diagnostic algorithms [6,13], as well as the neuropathology [9,11,13,20] have been described for human BoDV-1 encephalitis, very little is known about the immunology and potential immunopathological processes of this infection.

Here we analyzed serum, cerebrospinal fluid (CSF), and formalin-fixed paraffin-embedded (FFPE) brain autopsy samples from patients with fatal BoDV-1 encephalitis for inflammatory biomarker changes in order to shed more light on the immunology and (immune)pathogenesis of the disease. The analyses encompassed flow cytometry-based measurements of cytokine, chemokine, growth factor and other biomarker concentrations from serum and CSF, as well as comparable biomarker mRNA analyses and immunohistology examinations from autoptic brain tissue samples.

## Patients, materials and methods

### Patients and materials

A total of 11 patients were included in the study, all with acute, severe, and finally fatal BoDV-1 encephalitis acquired in virus-endemic regions of Germany [6,8,12–14] and one recent unpublished case). All cases fulfilled the published case definitions for confirmed BoDV-1 encephalitis [6]. Confirmation was achieved by positive quantitative reverse-transcription polymerase chain reaction (qRT-PCR) results for virus presence from either CSF, brain biopsy, or brain autopsy tissue in the different cases ([6,8,12–14]; in CSF from the unpublished case). From 9 patients, only serum and CSF samples were available. From one patient (patient 3), only FFPE brain autopsy samples could be retrieved. From another patient (patient 11), serum, CSF and FFPE brain autopsy samples were analyzed. Duration of disease from symptom onset until death was 3–8 weeks (Table 1). Sex and age were not disclosed in some patients due to local ethics regulations. Two post solid organ transplant patients were among the individuals included in the study (patient 4 and 5), receiving immunosuppressive treatment. Ethical clearance for this study was obtained from the Medical Board of Hamburg, no. PV5616.

**Table 1.** Characteristics of patients with fatal BoDV-1 encephalitis included in the study.

Patient number in this study	Year of symptom onset/Year of diagnosis	Sex/Age	Available patient sample material	Duration of disease (weeks)
1	2019/2019	F/11	S, CSF	4
2	2020/2020	M/79	S, CSF	5.5
3	2017/2020	M/59	BA	4
4	2001/2018	Not disclosed*	S, CSF	8
5	2008/2018	Not disclosed*	S, CSF	7
6	2016/2018	Not disclosed*	S, CSF	4
7	2017/2018	Not disclosed*	S, CSF	7
8	2019/2019	F/55	S, CSF	3
9	2020/2020	Not disclosed*/71	S, CSF	4
10	2020/2021	M/65	S, CSF	6
11	2021/2021	M/79	S, CSF, BA	6.5

Abbreviations: S, serum; CSF, cerebrospinal fluid; BA, formalin-fixed brain autopsy material.

*According to local ethics committee regulations.

### Measurement of cytokine, chemokine, growth factor and other biomarker concentrations in serum and cerebrospinal fluid

#### Samples and statistics

Serum and CSF samples were stored at −70°C until biomarker analyses were performed. Biomarker concentrations were analyzed by LEGENDplex (BioLegend, Fell, Germany) from all available sera and CSF at different time points after symptom onset. For analysis, results from sera of BoDV-1 patients were compared to sera of ten healthy uninfected blood donors as controls; results from CSF of BoDV-1 patients were compared to reference values from the literature [21–27]. In addition, to visualize mediator changes over time in an accumulated fashion in our patients with individual long disease courses, the duration of each patient’s illness was split in two equally long periods of time, i.e. two halves. Thus, for example, when a patient had a total disease duration of 4 weeks, the first half comprises the first two weeks of illness, and the second half comprises the last two weeks of illness. The respective sample analysis results were assigned to either of these disease duration halves (“phases”) depending on the sampling date. Statistical interpretation was performed with GraphPad Prism 7 (GraphPad Software Inc., La Jolla, CA). For comparison between two analyzed groups, the Mann–Whitney test was used; for comparison between three or more analyzed groups, the Kruskal–Wallis test with subsequent Dunn’s multiple comparisons test was used. Data of the two immunosuppressed patients (patients 4 and 5) were not included in the statistical analysis.

#### Mediator detection limits

The standard detection limits of the LEGENDplex assay for the analyzed biomarkers were as follows in alphabetical order: arginase (3.01 pg/mL), basic fibroblast growth factor (FGFb, 40.3 pg/mL), beta-nerve growth factor (β-NGF, 0.5 pg/mL), brain-derived neurotrophic factor (BDNF, 5.9 pg/mL), CC-chemokine ligand 2 (CCL-2, N/A), CCL-3 (4.1 pg/mL), CCL-4 (4.2 pg/mL), CCL-5 (83.3 pg/mL), CCL-17 (1.8 pg/mL), chemokine (C-X3-C motif) ligand 1 (CX3CL1, 184.4 pg/mL), C-X-C motif chemokine 10 (CXCL-10, N/A), granulocyte colony-stimulating factor (G-CSF, 23.6 pg/mL), granulocyte-macrophage colony-stimulating factor (GM-CSF, 22.7 pg/mL), interferon-α (IFNα, 19.5 pg/mL), interferon-γ (IFNγ, 5.1 pg/mL), interleukin (IL) 1β (5.3 pg/mL), IL-2 (3.2 pg/mL), IL-4 (4.5 pg/mL), IL-5 (4.0 pg/mL), IL-6 (5.2 pg/mL), IL-8 (N/A), IL-9 (4.9 pg/mL), IL-10 (3.9 pg/mL), IL-12p40 (0.9 pg/mL), IL-12p70 (19.5 pg/mL), IL-13 (5.1 pg/mL), IL-17A (5.0 pg/mL), IL-17F (3.7 pg/mL), IL-18 (2.0 pg/mL), IL-21 (3.9 pg/mL), IL-22 (2.0 pg/mL), IL-23 (1.2 pg/mL), platelet-derived growth factor BB (PDGF-BB, 29.6 pg/mL), soluble receptor for advanced glycation end products (sRAGE, 15.5 pg/mL), soluble triggering receptor expressed on myeloid cells 1 (sTREM-1, 12.7 pg/mL), sTREM-2 (N/A), transforming growth factor beta 1 (TGF-β1, 3.1 pg/mL), tumour necrosis factor (TNF, 5.1 pg/mL), and vascular endothelial growth factor (VEGF, 31.4 pg/mL).

### Molecular detection of cytokine, chemokine and other biomarker mRNA in FFPE brain tissue samples

#### Tissues, RNA extraction and PCRs

RNA from several brain regions (frontal, temporal, parietal and occipital cortex; hippocampus, basal ganglia, thalamus, pons, cerebellum) was extracted as described elsewhere [28] using the RNeasy FFPE Kit (Qiagen, Hilden, Germany). RNA yield was measured using the Qubit RNA HS Assay Kit (Invitrogen-Thermo Fisher Scientific, Waltham, MA, USA) following the manufacturer’s instructions. RNA quality was checked regarding purity (260/280 nm) and contamination (260/230 nm) using a NanoDrop 2000c spectrophotometer (Thermo Fisher Scientific). To ensure RNA quality, DNase digestion was performed as part of the RNA purification using the RNeasy FFPE Kit. Additionally, patient samples were tested in reference gene qPCR (see below) before proceeding with further analysis. In case the reference gene could not be detected, samples were excluded. Pooled RNA from the brains of five donors (BioChain, Newark, CA, USA) was used as a reference sample. Reverse transcription was performed using the High-Capacity cDNA Reverse Transcription Kit or the High Capacity RNA-to-cDNA-Kit (both from Applied Biosystems-Thermo Fisher Scientific). All biomarker qPCRs were performed with technical duplicates, using the Taqman Fast Advanced MasterMix (Applied Biosystems-Thermo Fisher Scientific). Due to the very limited amount of patient samples, the use of 3 or more technical replicates was unfortunately not possible. Efficiencies for qPCR assays were analyzed by testing the qPCR of each gene using a dilution series of the control sample (at least 3 dilution steps). Efficiency calculation was performed using the LightCycler 480 software (Release 1.5.1.62; Roche, Basel, Switzerland) and included in the respective relative gene expression analysis (efficiencies of 1.963–2.296; ideal value: 2.0).

qPCR for BoDV-1 was performed as described elsewhere [7,8]. BoDV-1 copy numbers were calculated by using the standard curve of the titrated synthetic positive control oligonucleotide with known molarity and copy number. RNA concentration was measured by Qubit and the viral copy number was calculated per ng sample RNA.

#### Biomarker gene analyses

cDNAs were analyzed for relative gene expression of human arginase 1 (ARG1), fibroblast growth factor (FGF1), glial fibrillary acidic protein (GFAP), IFNG, IL1RN, IL4, IL9, IL10, IL12B, IL23A, lipocalin 2 (LCN2), mannose receptor C-type 1 (MRC1), inducible nitric oxide synthase 2 (NOS2), resistin-like beta (RETNLB), and TGFB1 by TaqMan Assays (Applied Biosystems). Expression of genes for C1q (C1QA), C3, CCL2, CCL5, CXCL8, CXCL10, frizzled class receptor 1 (FZD1), insulin-like growth factor 1 (IGF1), IL1A, IL1B, IL6, IL13, tissue metallopeptidase inhibitor 1 (TIMP1), TNF and triggering receptor expressed on myeloid cells 2 (TREM2) was analyzed using self-designed primers and probes supplied by Biomers.net GmbH (Ulm, Germany; Online Appendix Table 1). Relative gene expression of biomarker mRNA was analyzed with an efficiency-corrected model for one sample [29], based on the delta-ct of the patient sample in correlation to the delta-ct of the healthy control.

#### Reference gene selection

For the potential references genes (ACTB, EEF2, GAPDH, NDUGB1, NDUFB4, PEA15 and PPA1), primers and probes were also self-designed (Biomers.net; Online Appendix Table 1). NDUFB4 and NDUFB1 are known to best meet published selection criteria for reference gene expression studies of human brain tissue [30]. Additionally, ACTB, EEF2, GAPDH, as well as PEA15 and EEF2 were selected from the online database for housekeeping genes and reference gene candidates (housekeeping.unicamp.br). Reference gene validation was performed using human astrocyte, microglia and microvascular brain endothelial cell lines (Applied Biological Materials, Richmond, Canada) kept in RPMI medium (PAN-Biotech, Aidenbach, Germany) and sampled at a minimum of four time points. Therefore, RNA was isolated using the RNeasy Kit (Qiagen), and reverse transcription was performed as described above. Genes were analyzed for their expression stability over time within each cell line as well as between the cell lines using NormFinder [31]. The reference gene should have the lowest possible intragroup variation (variation within samples of the same origin taken at different time points) as well as intergroup variation (variation within samples of different origin).

### Immunohistology of biomarkers

Available FFPE brain tissue samples were processed for routine histology with haematoxylin and eosin stains. Immunohistology was performed for biomarkers that were found to be detectable by the preceding molecular analysis (see legend of Figure 4). After pretreatment with buffers containing EDTA (pH 9) or citrate (pH 5 or 6) and endogenous peroxidase blocking, the FFPE tissue sections were incubated with the respective antibodies in 1% bovine serum albumin (BSA; Sigma-Aldrich/Merck, Taufkirchen, Germany) at room temperature overnight. This step was followed by incubation with the DCS-AEC 2 Component Detection Kit and 3-amino-9-ethylcarbazole substrate (DCS-diagnostics, Hamburg, Germany) for immunoperoxidase staining, and the DCS AP Detection Kit and new fuchsine substrate (DCS-diagnostics) for immunophosphatase staining. As negative control, the respective primary antibody was omitted.

## Results

### Cytokine, chemokine, growth factor and other biomarker concentrations in serum and cerebrospinal fluid

#### Differences of median biomarker levels between BoDV-1 encephalitis patients and controls

**Figure 1.**
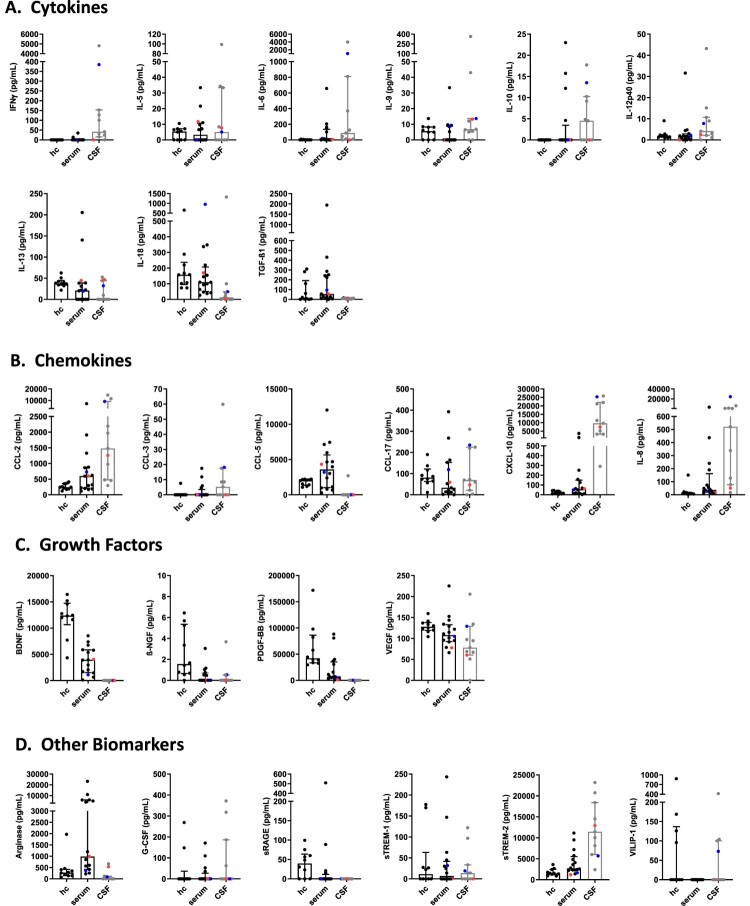
Cytokine, chemokine, growth factor and other biomarker changes in serum and cerebrospinal fluid of patients with BoDV-1 encephalitis. Serum (black) and CSF (grey) samples from ten BoDV-1 encephalitis patients and serum samples from ten healthy controls (hc) were analyzed in parallel for cytokines (panel A), chemokines (panel B), growth factors (panel C) and other biomarkers (panel D) by bead-based LEGENDplex assay. Results of two immunosuppressed patients are highlighted, patient 4 in red and patient 5 in blue. See Table 2 for median measurements of patient biomarkers and healthy controls in serum (including *p* values and confidence intervals) and Table 3 for median measurements of patient biomarkers (including confidence intervals) and reference values of biomarkers in CSF. Data are shown as median with interquartile range. β-NGF, beta-nerve growth factor; BDNF, brain-derived neurotrophic factor; CCL, CC-chemokine ligand; CXCL, C-X-C motif chemokine; G-CSF, granulocyte colony-stimulating factor; IFN, interferon; IL, interleukin; PDGF-BB, platelet-derived growth factor BB; sRAGE, soluble receptor for advanced glycation end products; sTREM, soluble triggering receptor expressed on myeloid cells; TGF, transforming growth factor; VEGF, vascular endothelial growth factor; VILIP, visinin-like protein.

In serum, levels of the cytokines IL-6 and TFG-β1 demonstrated an increase in BoDV-1 patients when compared to healthy controls; in contrast, levels of IL-5, IL-13 and IL-18 decreased. Concentrations of IL-12p40 were identical between patients and controls, while levels of IFNγ, IL-9 and IL-10 in patients were below the detection limits (Figure 1(A), Table 2). In CSF, levels of IFNγ and IL-6 displayed a marked increase in BoDV-1 patients, whereas concentrations of IL-10 were within reference range (Figure 1(A), Table 3). No CSF reference values could be found for IL-5, IL-9, IL-12p40, IL-13, IL-18 and TGF-β1 in the literature.

**Table 2.** Concentrations of cytokines, chemokines, growth factors and other biomarkers in sera of BoDV-1 encephalitis patients and healthy controls.

Biomarker	Median concentration in sera of BoDV-1 encephalitis patients (pg/mL) [95% CI]			Median concentration in sera of controls (pg/mL) [95% CI]
	Total	First phase of illness	Second phase of illness	
**Cytokines**	** **	** **	** **	** **
IFNγ	BDL [BDL]	BDL [BDL]	BDL [BDL-17.6]	BDL [BDL]
IL-5	BDL [BDL-11.1]	BDL [BDL]	6.6 [BDL-27.5]	5.3 [BDL-7.1]
IL-6	12.2* [BDL-163.8]	BDL [BDL]	102.1* [BDL-333.5]	BDL [BDL-5.8]
IL-9	BDL [BDL-9.3]	BDL [BDL]	BDL [BDL-33.4]	5.6 [BDL-8.5]
IL-10	BDL [BDL-12.2]	BDL [BDL]	BDL [BDL-17.6]	BDL [BDL]
IL-12p40	1.8 [BDL-4.5]	1.1 [1.1–1.1]	1.9 [BDL-31.6]	1.8 [1.1–2.8]
IL-13	21.0* [BDL-39.1]	15.0 [BDL-30.1]	20.2 [BDL-172.7]	37.4 [32.2–50.7]
IL-18	104.4 [42.4–219.1]	45.3 [37.5–53.0]	132.0 [36.6–348.3]	157.6 [73.4–288.8]
TGF-β1	44.1 [7.9–287.2]	127.2 [7.9–246.6]	55.4 [6.5–1941.0]	13.9 [BDL-283.5]
**Chemokines**	** **			
CCL-2	460.0 [189.4–1327.0]	331.7 [292.9–370.5]	543.8 [166.6–3911.0]	243.2 [171.2–368.8]
CCL-3	BDL [BDL-6.0]	BDL [BDL-5.9]	BDL [BDL-17.5]	BDL [BDL]
CCL-5	3503.0 [795.7–7141.0]	1843.0 [795.7–2890.0]	4689.0* [821.9–**12014.0]**	2054.0 [1190.0–2218.0]
CCL-17	25.2 [4.0–164.2]	78.4 [4.0–152.8]	22.5 [BDL-392.2]	81.0 [56.8–137.1]
CXCL-10	56.6 [11.2–250.1]	24.6 [13.1–40.0]	126.0* [11.2–**1836.0]**	20.0 [8.5–42.2]
IL-8	44.9*** [17.0–**240.9]**	18.1 [13.3–22.9]	112.7** [26.5–**3330.0]**	9.5 [6.0–23.3]
**Growth Factors**	** **			
BDNF	3935.0**** [1452.0–6742.0]	3861.0 [1669.0–6053.0]	3452.0** [374.3–**8535.0]**	12382.0 [7722.0–15217]
β-NGF	BDL** [BDL-1.0]	BDL [BDL-0.5]	0.5 [BDL-3.0]	1.6 [0.5–5.7]
PDGF-BB	9179.0** [5140.0–**40046.0]**	19886.0 [6177.0–33595.0]	11177.0 [2075.0–84380.0]	42119.0 [32664.0–97819.0]
VEGF	112.8 [91.8–143.5]	94.9 [94.8–95.0]	124.4 [93.8–225.2]	127.6 [116.1–141.4]
**Others**	** **			
Arginase	1200.0** [417.9–**6070.0]**	395.5 [223.1–567.9]	4970.0** [429.3–**23248.0]**	274.4 [138.3–456.2]
G-CSF	BDL [BDL-52.7]	BDL [BDL]	BDL [BDL-140.4]	BDL [BDL-148.0]
sRAGE	BDL [BDL-21.1]	BDL [BDL]	BDL [BDL-509.1]	39.5 [BDL-75.3]
sTREM-1	BDL [BDL-63.1]	BDL [BDL]	37.7 [BDL-146.9]	BDL [BDL-169.6]
sTREM-2	3199.0** [2325.0–**7178.0]**	4297.0 [2328.0–6267.0]	3570.0* [2207.0–**7627.0]**	1524.0 [930.7–2705.0]

BDL = below detection limit.

CI =  confidence interval.

Sera of BoDV-1 patients (Total) were compared to healthy serum controls; Statistical analyses were performed with Mann-Whitney test.

Sera of BoDV-1 patients from the two phases were compared with each other and the healthy serum control; Statistical analyses were performed with Kruskal-Wallis test and subsequent Dunn’s multiple comparisons test.

Statistically significant differences were found between the phases and the control, as indicated by the asterisks. No significant differences were detected between the two phases of illness.

Asterisks indicate statistically significant differences (*p < 0.05, **p < 0.01, ***p < 0.001, ****p < 0.0001). Significant changes are shown in bold face.

The two immunosuppressed patients were not included in the statistical analysis.

**Table 3.** Concentrations of cytokines, chemokines, growth factors and other biomarkers in CSF of BoDV-1 encephalitis patients and of controls from the literature.

Biomarker	Median concentration in CSF of BoDV-1 encephalitis patients (pg/mL) [95% CI]			Concentration in CSF of controls (pg/mL), (literature reference)
	Total	First phase of illness	Second phase of illness	
**Cytokines**	** **	** **	** **	** **
IFNγ	41.5 [15.7–153.5]	20.8 [BDL-41.5]	92.8 [24.0–4804.0]	<5.0 (21)
IL-5	BDL [BDL-34.3]	4.2 [BDL-8.4]	8.3 [BDL-98.8]	N/A
IL-6	88.5 [11.7–810.2]	22.0 [BDL-44.1]	229.5 [11.7–3984.0]	<12.5 (21)
IL-9	6.5 [BDL-43.0]	188.4 [12.0–364.9]	6.1 [BDL-24.0]	N/A
IL-10	4.5 [BDL-10.3]	BDL [BDL]	5.0 [BDL-17.7]	<5.0 (21)
IL-12p40	4.2 [1.2–15.1]	BDL [BDL-1.2]	7.9 [2.2–43.2]	N/A
IL-13	BDL [BDL-44.5]	22.3 [BDL-44.5]	BDL [BDL-52.7]	N/A
IL-18	6.4 [BDL-100.0]	BDL [BDL]	31.5 [BDL-1329.0]	N/A
TGF-β1	9.9 [3.3–13.3]	6.8 [BDL-13.5]	9.7 [5.4–12.4]	N/A
**Chemokines**	** **			
CCL-2	1258.0 [457.8–11563.0]	1223.0 [477.4–1968.0]	1709.0 [297.8–14708]	125–375 (21)
CCL-3	5.3 [BDL-17.6]	BDL [BDL-5.3]	4.2 [BDL-59.8]	<2.5 (21)
CCL-5	BDL [BDL]	BDL [BDL]	BDL [BDL-2702.0]	N/A
CCL-17	69.9 [3.6–224.8]	11.7 [BDL-21.3]	130.5 [3.6–224.8]	N/A
CXCL-10	9631.0 [3225.0–22004.0]	2753.0 [290.3–5216.0]	16251.0 [3238.0–25838.0]	median: 9 (23)
IL-8	521.1 [77.3–1314.0]	396.0 [77.3–714.8]	513.7 [17.6–6142.0]	12.5–25 (21)
**Growth Factors**	** **			
BDNF	BDL [BDL-10.6]	BDL [BDL]	BDL [BDL-26.1]	median: 1.6 (27); 0–40 (25)
β-NGF	BDL [BDL-0.5]	BDL [BDL]	BDL [BDL-3.7]	N/A
PDGF-BB	BDL [BDL]	BDL [BDL]	BDL [BDL]	N/A
VEGF	78.1 [50.9–134.2]	48.7 [BDL-97.3]	86.4 [58.9–205.5]	2.73 (24)
**Others**	** **			
Arginase	BDL [BDL-117.0]	58.5 [BDL-117.0]	BDL [BDL-666.2]	<6400 (26)
G-CSF	BDL [BDL-318.0]	31.4 [BDL-62.8]	93.2 [BDL-371.4]	N/A
sRAGE	BDL [BDL]	BDL [BDL]	BDL [BDL]	N/A
sTREM-1	13.7 [BDL-96.8]	BDL [BDL]	13.9 [BDL-121.8]	157.72 ± 7.84 (22)
sTREM-2	11441.0 [6009.0–20737.0]	5254.0 [2384.0–8123.0]	15331.0 [6009.0–23172.0]	0–10,000 (21)

BDL = below detection limit.

N/A = not available in the literature.

CI = confidence interval.

CSF of BoDV-1 patients from the two phases were compared with each other. Statistical analyses were performed with Mann-Whitney test, and no significant differences were detected between the phases.

No statistical analyses were performed between the two phases and the reference values from the literature.

The two immunosuppressed patients were not included in the statistical analysis.

In serum, levels of the chemokines CCL-2, CCL-5, CXCL-10 and IL-8 showed a marked increase in BoDV-1 patients. In contrast, concentrations of CCL-17 displayed a prominent decrease, while CCL-3 concentrations were below the detection limit in both groups (Figure 1(B), Table 2). In CSF, levels of CCL-2, CXCL-10 and IL-8 demonstrated a striking increase in BoDV-1 patients; this increase was less for CCL-3 (Figure 1(B), Table 3). No CSF reference values were found for CCL-5 and CCL-17 in the literature.

In serum, decreased levels of the growth factors BDNF, β-NGF, PDGF-BB and VEGF were noted in BoDV-1 patients when compared to controls (Figure 1(C), Table 2). In CSF, concentrations of BDNF, β-NGF and PDGF-BB were below the detection limit in BoDV-1 patients. In contrast, VEGF levels demonstrated a prominent increase (Figure 1(C), Table 3). No CSF reference values could be found for β-NGF and PDGF-BB in the literature.

In serum, concentrations of the other biomarkers arginase and sTREM2 were elevated in BoDV-1 patients, while the levels of sRAGE were decreased. Levels of G-CSF and sTREM-1 were below the detection limits in patients and controls (Figure 1(D), Table 2). In CSF, arginase concentrations were below the detection limits in BoDV-1 patients, while sTREM-2 levels were slightly increased, and sTREM-1 levels showed a marked decrease (Figure 1(D), Table 3). No CSF reference values could be retrieved for G-CSF and sRAGE in the literature.

In the two immunosuppressed patients, no specific common pattern could be discerned; in patient 5 however, many cytokines and chemokines showed high concentrations.

Serum and CSF concentrations of FGFb, CCL-4, CX3CL1, GM-CSF, IFNα, IL-1β, IL-2, IL-4, IL-12p70, IL-17A, IL-17F, IL-21, IL-22, IL-23 and TNF were either similar between patients and controls or below the detection limits (data not shown).

#### Changes of median biomarker levels during different phases of BoDV-1 encephalitis

**Figure 2.**
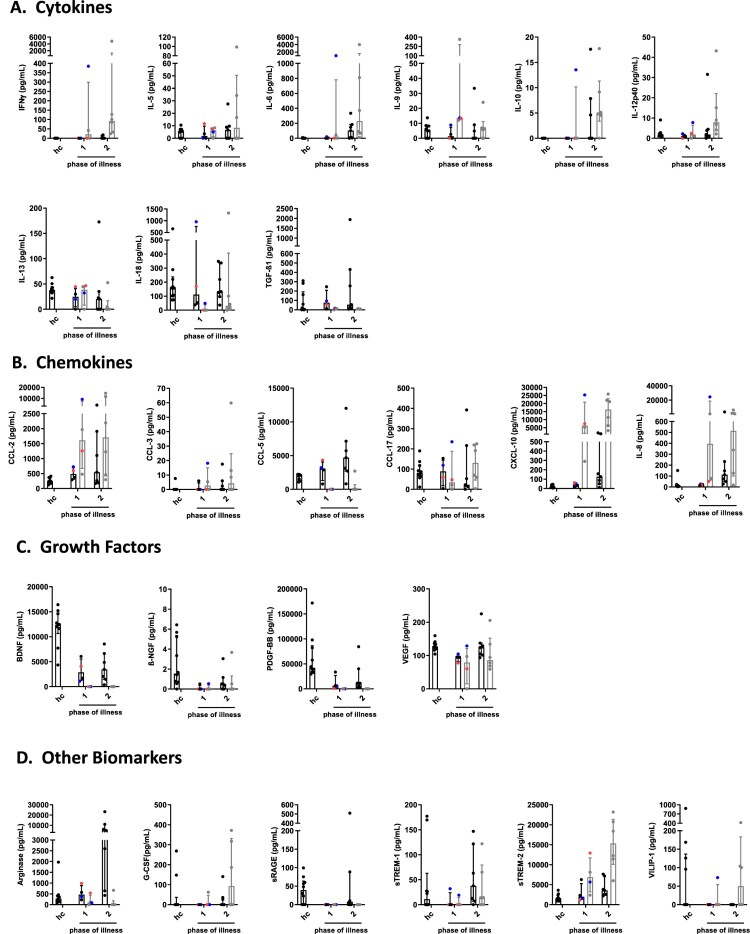
Cytokine, chemokine, growth factor and other biomarker changes in serum and cerebrospinal fluid of patients with BoDV-1 encephalitis in two phases of illness. Serum (black) and CSF (grey) samples from ten BoDV-1 encephalitis patients and serum samples from ten healthy controls (hc) were analyzed in parallel for cytokines (panel A), chemokines (panel B), growth factors (panel C) and other biomarkers (panel D) by bead-based LEGENDplex assay. Results of two immunosuppressed patients are highlighted, patient 4 in red and patient 5 in blue. See Table 2 for median measurements of patient biomarkers and healthy controls in serum (including *p* values and confidence intervals) and Table 3 for median measurements of patient biomarkers (including confidence intervals) and reference values of biomarkers in CSF. For analysis, sampling dates of the patients were assigned to different phases of the illness after symptom onset. Therefore, the individual duration of illness in each patient was split in two equally long time periods (halves). Data are shown as median with interquartile range. β-NGF, beta-nerve growth factor; BDNF, brain-derived neurotrophic factor; CCL, CC-chemokine ligand; CXCL, C-X-C motif chemokine; G-CSF, granulocyte colony-stimulating factor; IFN, interferon; IL, interleukin; PDGF-BB, platelet-derived growth factor BB; sRAGE, soluble receptor for advanced glycation end products; sTREM, soluble triggering receptor expressed on myeloid cells; TGF, transforming growth factor; VEGF, vascular endothelial growth factor; VILIP, visinin-like protein.

When different phases were analyzed, serum concentrations of the cytokines IFNγ, IL-9 and IL-10 were below the detection limit in BoDV-1 patients, while levels of IL-5, IL-6, IL-12p40, IL-13 and IL-18 increased from the first to the second phase of the disease. Decreases of TGF-β1 levels were detected from the first to the second phase of illness. For TGF-β1, concentrations were higher in both phases than in controls (Figure 2(A), Table 2). In CSF, concentrations of IFNγ, IL-5, IL-6, IL-10, IL-12p40, IL-18 and TGF-β1 showed an increase from the first phase of illness to the second phase. Levels of IFNγ and IL-6 exceeded the reference values from the literature in both analyzed phases by far. In contrast, levels of IL-9 and IL-13 decreased from the first phase of the disease to the second (Figure 2(A), Table 3).

In serum, levels of the chemokines CCL-2, CXCL-10 and IL-8 were increased in BoDV-1 patients in the first phase of the disease compared to controls, reaching maximum concentrations in the second phase. CCL-5 and CCL-17 expressions were similar in the first phase of illness and the control group; CCL-5 increased and CCL-17 decreased in the second phase of illness. CCL-3 levels were below the detection limit in either phase of the disease and in controls (Figure 2(B), Table 2). In CSF, increasing levels of CCL-2, CCL-3, CCL-17, CXCL-10 and IL-8 were detectable from the first to the second phase of illness; CCL-2, CXCL-10 and IL-8 concentrations were far above the reference values from the literature in both phases of the disease. CCL-5 expression was below the detection limit in either phase (Figure 2(B), Table 3).

In serum, concentrations of the growth factors BDNF, β-NGF, PDGF-BB and VEGF in BoDV-1 patients were decreased in both phases of the disease in comparison to controls (Figure 2(C), Table 2). In CSF, levels of BDNF, β-NGF and PDGF-BB were below the detection limit in either phase of illness, whereas VEGF concentrations were elevated in both phases (Figure 2(C), Table 3).

In serum, the other biomarkers arginase and sTREM-1 showed increasing levels in BoDV-1 patients from the first to the second phase of illness, while sTREM-2 decreased. Concentrations of G-CSF and sRAGE were below the detection limit in either phase (Figure 2(D), Table 2). In CSF, arginase levels were only detectable in the first phase, and sRAGE in neither phase. G-CSF, sTREM-1 and sTREM-2 increased during the second phase. Levels of sTREM-2 reached concentrations above the reference value from the literature (Figure 2(D), Table 3).

From the two immunosuppressed patients samples were available for phase one only, showing similar results as above in the comparison of BoDV-1 patients with healthy controls.

Serum and CSF concentrations of FGFb, CCL-4, CX3CL1, GM-CSF, IFNα, IL-1β, IL-2, IL-4, IL-12p70, IL-17A, IL-17F, IL-21, IL-22, IL-23 and TNF were either similar between patients and controls or below the detection limits (data not shown).

### Molecular detection of cytokine, chemokine and other biomarker mRNA in FFPE brain tissue samples

Of the two cases from whom autopsy FFPE material was available, RNA of sufficient quantity and quality could be extracted from three samples of each patient. The samples included hippocampus, basal ganglia and thalamus of patient 3, and hippocampus, basal ganglia and the occipital lobe of patient 11. Using NormFinder, NDUFB4 could be identified as the most stable expressed gene (stability value 0.082) based on the overall lowest intragroup and intergroup variation and was therefore used as the reference gene for relative expression analysis (Online Appendix Table 2).

**Figure 3.**
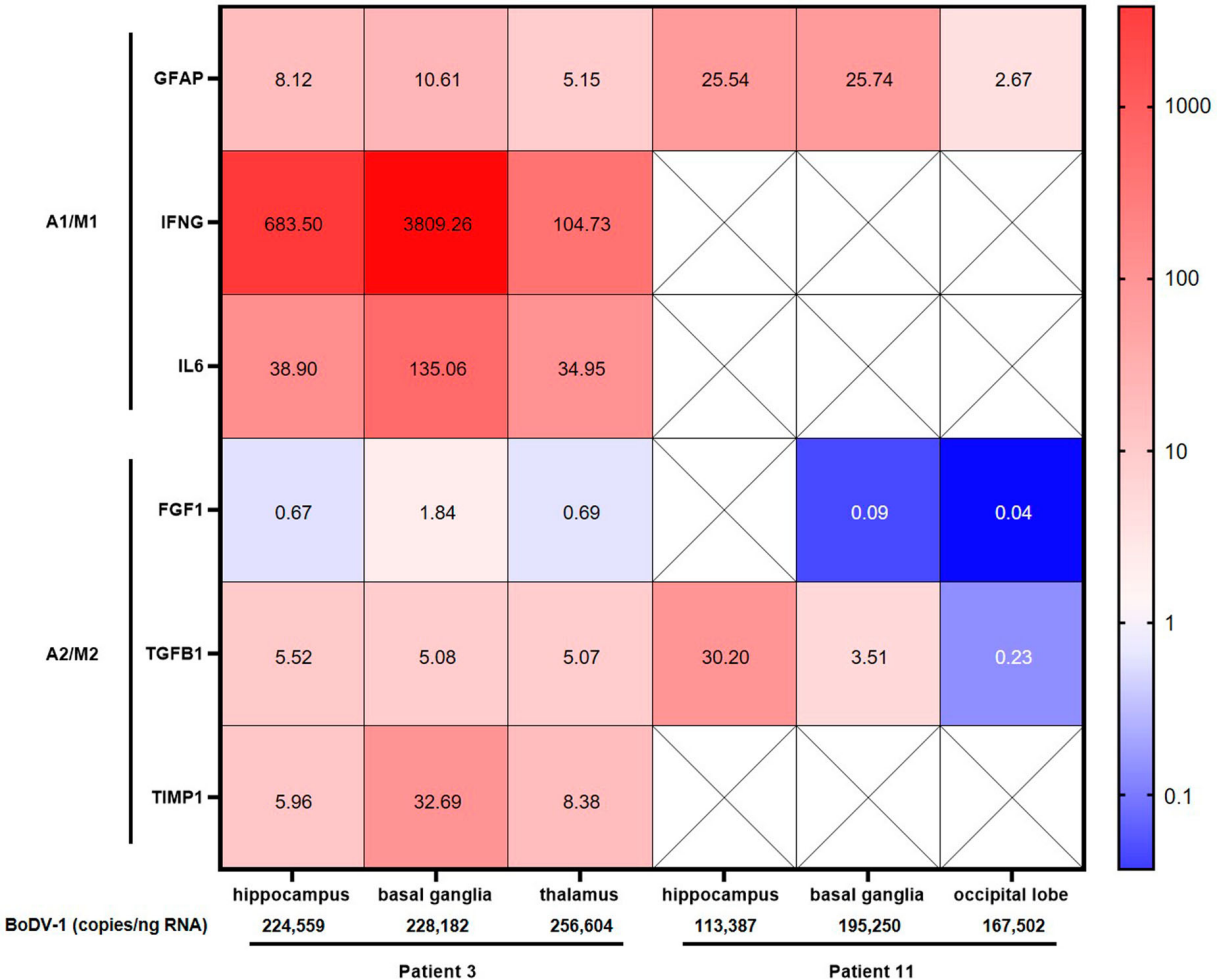
Expression of inflammatory and anti-inflammatory biomarkers in fixed brain tissue of patients with BoDV-1 encephalitis. Relative expression of biomarker gene mRNA was detected in FFPE brain tissue of three different brain regions from two patients. Relative expression is shown as fold change to reference sample. Colour gradient is presented as log10. Astrocyte and microglia pro-inflammatory/classical activation markers (A1/M1) showed an increased expression in patient 3, with strongest increase in pro-inflammatory IFNG, followed by IL6 and GFAP. Anti-inflammatory/regulatory activation state markers (A2/M2) TGFB1 and TIMP1 showed a slight increase, whereas no change was detected in FGF1 expression. In comparison to hippocampus and thalamus, basal ganglia demonstrated the strongest increase in gene expression in patient 3. Less data could be acquired from patient 11. Crossed-out squares label markers that were not detectable by qPCR. An increased GFAP expression, as well as decreased FGF1 expression, is similar to patient 3. In addition, BoDV-1 copy numbers are shown, demonstrating higher viral loads in patient 3 than in patient 11.

Overall, the qPCRs targeting cytokine, chemokine and other biomarker genes yielded positive results from the three brain regions with sufficient RNA quality of the two patients only for GFAP, IFNG, IL6, TGFB1 and TIMP1. Relative gene expression of these markers in FFPE material from patient 3 showed an increase in some astrocyte and microglia pro-inflammatory markers, with the strongest increase in IFNG, followed by IL6 and GFAP (Figure 3). The anti-inflammatory activation state markers TGFB1 and TIMP1 showed a slight increase, whereas, except for the basal ganglia, a decreased FGF1 expression was detected. In comparison to hippocampus and thalamus, the basal ganglia of patient 3 demonstrated the overall strongest increase in gene expression. Less positive results could be obtained from RNA of patient 11. Nevertheless, the increased GFAP expression, as well as the decreased FGF1 expression of patient 11, correlated with data from patient 3.

BoDV-1 copy numbers ranged from 113,387–256,604/ng RNA with higher viral loads in patient 3 than in patient 11. Highest viral copy numbers were found in the thalamus of patient 3 (Figure 3).

### Immunohistology of biomarkers in FFPE brain tissue samples

**Figure 4.**
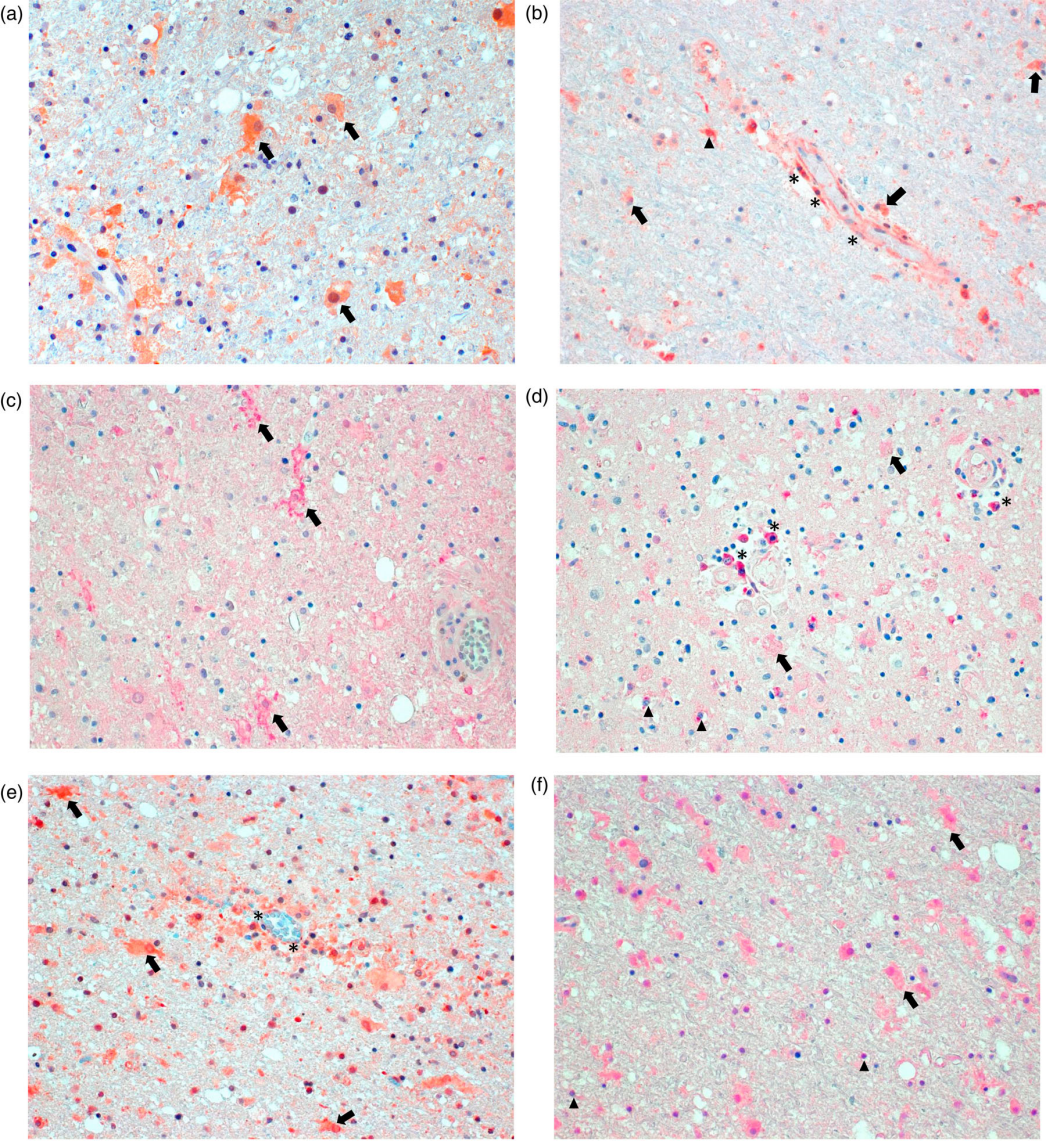
Immunohistology for molecularly detectable biomarkers in the brain of patients with BoDV-1 encephalitis. Panel A: Immunoperoxidase staining for glial fibrillary acidic protein (GFAP, 1:100; Zytomed Systems, Bargteheide, Germany; with citrate pretreatment), demonstrating activated, bizarre and enlarged astrocytes (arrows). Panel B: Immunoperoxidase staining for interferon-γ (IFNγ; 1:300; Abcam, Cambridge, UK; with citrate pretreatment), showing positivity in endothelial cells (asterisks), and most likely in microglia (arrowheads) and astrocytes (arrows) perivascularly. Panel C: Immunophosphatase staining for interleukin 6 (IL-6; Biosource-Thermo Fisher Scientific; with EDTA pretreatment), demonstrating ramified activated microglia (arrows). Panel D: Immunophosphatase staining for fibroblast growth factor-1 (FGF-1; LifeSpan BioSciences, Huissen, the Netherlands; with EDTA pretreatment), showing positivity in perivascular brain cells (asterisks), oligodendrocytes (arrowheads) and weakly in enlarged astrocytes (arrows). Panel E: Immunoperoxidase staining for transforming growth factor-β (TGF-β; DCS-diagnostics; with citrate pretreatment), demonstrating positivity in endothelial cells (asterisks) and mainly in activated astrocytes (arrows). Panel F: Immunophosphatase staining for tissue inhibitor of metalloproteinase 1 (TIMP1; Biosource-Thermo Fisher Scientific; with citrate pretreatment), showing positivity in activated and enlarged astrocytes (arrows) and in oligodendrocytes (arrowheads). All panels show basal ganglia sections with an original magnification of ×200. Symbols show a few examples of cell types only.

Immunostaining for biomarkers in brain tissue areas found to show detectable results in the molecular detection assays of the two patients was positive for various cell types (Figure 4). Staining for GFAP showed activated astrocytes throughout (Figure 4(A)), whereas staining for IFNγ was positive primarily around blood vessels, indicating production in endothelial cells, and most likely in perivascular microglia and astrocytes (Figure 4(B)). Staining for IL-6 was positive in activated microglia (Figure 4(C)), while FGF was detected in perivascular brain cells, oligodendrocytes and weakly in enlarged astrocytes (Figure 4(D)). TGF-β could be demonstrated in endothelial cells, activated astrocytes, and in a few microglia (Figure 4(E)). TIMP1 was detected in activated astrocytes and in oligodendrocytes (Figure 4(F)). Control sections omitting the primary antibody did not show any specific staining (not shown).

## Discussion

In this study, we demonstrate a marked immune activation in patients with severe and finally fatal BoDV-1 encephalitis. Our analysis using a broad range of flow cytometry-based biomarker detection assays, qRT-PCR-facilitated biomarker mRNA measurements and immunostaining show striking changes in serum, CSF, and brain tissue, respectively. Our investigation provides a glimpse on the rather complex changes of serum and CSF inflammatory changes during disease progression of fatal BoDV-1 encephalitis. The splitting in two phases of BoDV-1 encephalitis disease provided a more detailed insight into the immunopathology. In the BoDV-1 encephalitis patients, elevations of the pro-inflammatory cytokines IFNγ and IL-6 were shown, with a decrease of anti-inflammatory IL-13 and an upregulation of anti-inflammatory TGF-β1 levels. This mixed cytokine state is dominated by pro-inflammatory activation intensifying over time in phase 2 and partly counteracted by anti-inflammatory markers. This was paralleled by increased levels of various chemokines such as pro-inflammatory CCL-2 and CXCL-10 mainly in CSF of BoDV-1 patients, and most strikingly by the angiogenetic factor/neutrophil attractant IL-8, likely reflecting ongoing attraction of immune cells to the site of infection and further contributing to inflammation and tissue damage. Growth factor levels of BDNF, β-NGF, PDGF-BB were strikingly decreased in the patients. Only VEGF concentrations showed increases in CSF, likely reflecting tissue repair attempts in phase two of the disease. While the levels of the other biomarkers arginase and sTREM-2 increased from the first to the second phase of illness in serum, arginase levels in CSF remained within reference limits and sTREM-2 increased. Arginase induces polarization of microglia to an anti-inflammatory state and neuroprotection by reduction of nitric oxide (NO) production [34], whereas sTREMs promote microglial survival and stimulate the production of inflammatory cytokines [32,33]. Thus, the levels of these other biomarkers also contributed to the pro-inflammatory state in the CNS toward phase two of BoDV-1 encephalitis. In the two immunosuppressed patients (patients 4 and 5), no specific pro-inflammatory or anti-inflammatory pattern could be seen that would stand out as specific for both of them, but both of them had the longest duration of disease. In patient 5, many cytokines and chemokines showed high elevations; this patient might have been an individual high cytokine responder as seen in other diseases. Unfortunately, no samples were available from the second phase of disease in either of these two patients to further evaluate and compare the findings of the first phase. Testing of serum and CSF during meningitis and encephalitis is an emerging field. Many chemokines and cytokines were found to be elevated during bacterial and viral CNS infections as shown by others [21]; thus, the changes seen in our investigation might not be specific for BoDV-1 encephalitis. Similar to other, non-specified forms of viral meningitis [21], we observed IFNγ and chemokine elevations in the CSF. As the current and severe BoDV-1 encephalitis was the most prominent condition in the patients, we strongly assume that the changes of biomarkers are associated with the current BoDV-1 disease. We cannot exclude, however, that any underlying disease might have influenced the mediator changes in addition.

In autoptic brain tissue, representing the end of phase two of disease, only few biomarkers were detectable likely due to RNA degradation (see further below). Of those which could be successfully examined (GFAP, IFNG, IL6, FGF1, TGFB1 and TIMP1), especially IFNG and IL6 were upregulated when compared to healthy control brain tissue. These pro-inflammatory cytokines were also found to be upregulated in CSF during infection, and IL-6 also in serum. Immunostaining revealed IFNγ production in endothelial cells, microglia and astrocytes, while IL-6 production was shown in ramified activated microglia. In contrast, FGF1 mRNA levels were decreased when compared to healthy controls. This finding is in line with the decreased levels of most growth factors in serum and CSF during BoDV-1 encephalitis. Weak positivity was demonstrated in perivascular brain cells, oligodendrocytes and astrocytes by immunostaining. TGFB1 was moderately upregulated in brain tissue during the final stage of the infection when compared to controls, likely mirroring the elevated serum concentration of anti-inflammatory TGF-β1. Unfortunately, no CSF reference values were available. Production of TGF-β1 was shown in endothelial cells, activated astrocytes and in a few microglia. TIMP1 levels in tissue were also only moderately elevated; production was demonstrated in oligodendrocytes and in activated and enlarged astrocytes. TIMP1 is secreted by astrocytes in the early phase of inflammation and a neuroprotective attempt [35]. In astrocytes, our investigation showed also increased GFAP production, a general immunostaining hallmark of activation [36,37]. Such activated, bizarre and enlarged astrocytes were demonstrated in both VSBV-1 [19] and BoDV-1 encephalitis [9,11]. No obvious correlations between the BoDV-1 copy numbers and gene expressions for GFAP, FGF1 and TGFB1 were seen when both patients were compared. No correlations between viral copy numbers and mediator gene expressions were found either when the different brain areas were compared within one patient. However, higher viral loads were detected in patient 3, who had a shorter course of disease than patient 11. Also in the related human VSBV-1 encephalitis, higher viral loads were found to be associated with shorter disease duration and thus earlier death of the patients [19]. However, the relation of viral copy numbers to disease course and mediator gene expression is based on a few patients only and warrants further investigations.

Astrocytes account for roughly 30% of the cells in the CNS. Besides the long-known homeostatic functions of these cells (i.e. scar formation, repair of blood–brain-barrier, neurotrophic support, synaptic and extracellular fluid maintenance), there is mounting evidence for an important immunological role during inflammation which includes cytokine secretion [37–39]. Microglia represent 15% of the cells of the brain parenchyma, and, as macrophage-like cells of the CNS, are responsible for establishing innate immune responses to pathogens, clearance of pathogens and debris, and maintaining tissue homeostasis [40]. Differently activated microglia, such as pro-inflammatory M1 microglia and anti-inflammatory M2 microglia, induce at least two reactive astrocyte states during neuroinflammation (or vice versa) and form a functional axis [37,38,40,41]: A1 astrocytes are regarded as pro-inflammatory and neurotoxic, whereas A2 astrocytes are seen as anti-inflammatory and neurotrophic [35,37,38]. The concept has been adopted from the original M1/M2 macrophage paradigm which reflects immunoregulation outside the CNS. While the biomolecules C1q, C3, CCL-2, CCL-20, CCR-2, CXCL-10, IFNγ, IL-1α/β, IL-6, IL-12, IL-23, LCN2, NO/NOS2, TNF (not exhaustive) are A1/M1 activation markers, the biomolecules arginase, CCL-17, CCL-22, CCL-24, CXCL-4, FGF1, FIZZ1, IL-3, IL-4, IL-10, IL-13, IL-21, IL-33, MRC1, RETNLB, TGF-β1, TIMP1, VEGF (not exhaustive) reflect an A2/M2 state [34, 37–41]. In our study, astrocytes showed production of pro-inflammatory IFNγ and less so of anti-inflammatory TGF-β1 and TIMP1. In activated microglia, we demonstrated production of pro-inflammatory IFNy, IL-6 and anti-inflammatory TGF-β1, embedded in, and likely responsible for, a predominantly A1/M1 soluble cytokine milieu maintaining ongoing inflammation in the brain.

In animals, lesions in the CNS during natural Borna disease or experimental infection are caused by a T cell-mediated immunopathology [42,43]; symptoms begin with the appearance of CD4 and CD8 T cells in the brain. Although antiviral CD8 T cells recognize BoDV-1 antigen in neurons and astrocytes [44], the functional avidity of these cells is apparently down-modulated during BoDV-1 infection [45]. While cytotoxic CD8 T cells were experimentally shown to be induced by virus-specific CD4 T cells [46], the CD8 cells required IFNγ to clear the virus from the brain [47]. In our study IFNγ production was demonstrated in endothelial cells, astrocytes and microglia, and the biomarker was present in CSF. Likewise, in experimental rodent studies with BoDV-1, increased IFNγ mRNA concentrations were also seen [48,49]. In animals, BoDV-1 was shown to replicate initially in neurons and later in astrocytes [50]. In a recent study of human BoDV-1 pathology no virus RNA was seen in microglial cells [11]. *In vitro* experiments hint at an important role of astrocytes activating microglia during BoDV-1 infection which, in turn, produce IL-6 [51,52]. In our study, IL-6 production was also shown in microglia, as well as in serum and CSF. Similarly, increased IL-6 mRNA concentrations were seen in experimental rodent infections with BoDV-1 [48,49]. Besides such IFNγ and IL-6 pro-inflammatory cytokine responses in animals, which included also IL-1, IL-2 and TNF mRNA elevations, anti-inflammatory responses by IL-4 and TGF-β mRNA increases were seen [49]. In another rodent model, the administration of TGF-β led to a transient inhibition of symptomatic BoDV-1 disease, paralleled by a significant reduction of the inflammatory reaction in the brain with reduced CD4+ T cells and markedly decreased CD8+ cells [53]. While we did not detect IL-1, IL-2, TNF and IL-4 responses in our patient cohort, TGF-β1 responses were seen. In rodents, a shift from TH1 to TH2 cytokine responses was assumed based on IL-4 and TGF-β elevations during the course of experimental BoDV-1 infection which led from an acute encephalitis to a chronic persistent viral infection [49]. Such a shift from a corresponding A1/M1 to A2/M2 state in human BoDV-1 encephalitis was not that evident and only weak, and might therefore be a possible reason for the fatal outcomes of human infections. Moreover, similar to the generally decreased growth factor levels described here in the BoDV-1 patients, decreased mRNA concentrations of BDNF were seen in experimental rodent infections with BoDV-1 [48]. Besides the predominantly pro-inflammatory cytokine and chemokine milieu described here in acute human BoDV-1 encephalitis contributing to an assumed neurodamaging state, the impairment of normal astrocyte function may lead to excitotoxicity in neurons due to glutamate excess [42] and subsequent neuronal lesions. However, the impact of such astrocytic dysregulation warrants further investigation.

This study has several limitations. Our work demonstrates the difficulty in obtaining high-quality RNA for biomarker analysis from FFPE tissues. This is most probably due to the severe inflammation and tissue damage seen in BoDV-1 encephalitis and the *post mortem* tissue autolysis before fixation, complicated by the fixation process itself. The *post mortem* interval before fixation seems to be a key factor [54,55]. When compared to neuronal and glia gene expression, the housekeeping genes used as reference for RNA normalization, as in our case, are less affected [55]. We have therefore screened the respective tissues for measurably expressed housekeeping genes before analyzing the biomarker genes. In addition to these detrimental natural effects, the formalin fixation leading to nucleic acid cross-links renders RNA less accessible for PCR analysis [28]. It is likely that such analyses from fixed biopsies (rather than from fixed autopsy material) or rapidly fresh-frozen autopsy material will be of better quality and thus higher validity in future studies. As a further limitation of the study, only few patients were included in the analysis and not all materials were available from all individuals. Future studies will have to address the described immunopathological findings with more patients included. For some biomarkers no reference values for CSF were available in the literature, as hardly healthy subjects undergo lumbar puncture. Nonetheless, we here demonstrate an emerging picture of marked immunoactivation leading to complex inflammation and a neurodamaging state.

In conclusion, our data, though fragmentary, demonstrate an increasing immune activation from the initial phase to the last phase of acute BoDV-1 encephalitis. A pro-inflammatory state as part of the immuno-mediated pathology, shown by elevated respective cytokines, chemokines and other biomarkers, dominates during the course of human BoDV-1 encephalitis. This dysbalanced, pro-inflammatory state leads to the severe and mostly fatal outcome of human BoDV-1 encephalitis, and might be a key target for possible treatment attempts with immunomodulating or immunosuppressive drugs in addition to antivirals.

## Supplementary Material

Supplemental Material
